# Unveiling the hidden burden: Common mental disorders among women attending antenatal care in selected health facilities of Lusaka, Zambia

**DOI:** 10.1371/journal.pmen.0000614

**Published:** 2026-06-17

**Authors:** Choolwe Jacobs, Josephine Nkhowani, Dennis Ngosa, Kwame Shanaube, Ingvild Fossgard Sandøy

**Affiliations:** 1 Department of Epidemiology and Biostatistics, School of Public Health, University of Zambia, Lusaka Zambia; 2 Women in Global Health, Lusaka, Zambia; 3 Analysis Unit, Centre for Infectious Disease Research in Zambia, Lusaka, Zambia; 4 Zambart, Lusaka, Zambia; 5 Department of Global Public Health and Primary Care, Centre for International Health and Bergen Centre for Ethics and Priority Setting in Health, Bergen, Norway; University of Minnesota Twin Cities Department of Medicine, UNITED STATES OF AMERICA

## Abstract

Mental health disorders disproportionately affect women during pregnancy and postpartum, with high prevalence in low-income countries. Mental health disorders during the antenatal period can lead to serious complications for both mother and child, including preterm birth, developmental issues and postpartum depression. However, mental health screening remains neglected during the antenatal period in resource-limited settings. We investigated the prevalence and determinants of common mental disorders (CMD) among women attending antenatal care (ANC) in selected health facilities of Lusaka, Zambia. A cross-sectional study was conducted between 26^th^ April and 6^th^ July 2024 in two purposively selected health facilities in low socioeconomic areas of Lusaka. A systematic sample of pregnant women in the third-trimester attending ANC were invited to participate. Common mental disorders were defined using a cut-off score of ≥8 on the WHO Self-Reporting Questionnaire (SRQ)-20, administered through face-to-face interviews. Logistic regression models were employed to identify determinants of common mental disorders. Stata 18 SE was used for analysis. We included 331 participants who were predominantly young adults (50.5% aged 20–29), married/cohabiting (69%), and almost a quarter (23.9%) living with HIV. Most had unplanned pregnancies (58.6%), experienced pregnancy-related body changes (73.1%) and had delivery concerns (68.6%). The study found a high prevalence of common mental disorders (65.8%). Participants who were less than 20 years, primary level education, had history of complications during previous pregnancy, were worried about the birth process and reported unpleasant body changes due to pregnancy exhibited higher prevalence for common mental disorders. On the other hand, women with medium or high social connectedness had lower odds of common mental disorders. The high burden of common mental health disorders among pregnant women in Lusaka, Zambia, underscores the importance of integrating mental health screening and support into routine antenatal care, with targeted attention to high-risk groups such as adolescents.

## Introduction

Common mental disorders (CMD) are a significant public health concern globally. They disproportionately affect women, especially during pregnancy and the postpartum period [1,2]. Recent systematic reviews and meta-analyses indicate a prevalence of antenatal depression of 20.7% (any depression) and 15.0% (major depression) [3], and a postnatal depression prevalence of 17% [4]. In sub-Saharan Africa, the burden of mental disorders among pregnant women is high, affecting 1 in 4 perinatal women [5].

Mental health issues such as depression, anxiety, and stress during pregnancy may have profound consequences for maternal health, child development, and family well-being [6]. Poor mental health during the gestation period is associated with higher probability of preterm birth, preeclampsia, hypertension, diabetes, postpartum psychological complications, suicidal ideations and the baby having low birth weight [7,3]. Children born to mothers suffering from unmanaged mental health conditions during the antenatal period face an increased susceptibility to long-term behavioral and neurodevelopmental issues during childhood and adolescence [8].

Research has shown that the risk of symptoms of common mental disorders during pregnancy is typically associated with being single [9], unplanned or poorly timed pregnancies [10–12], gender based violence [13] and young age (15–24) [14,15]. Existing research suggests that pregnant and parenting adolescents are at greater risk of experiencing depressive symptoms than pregnant and postpartum adult women [15]. The psychological and physiological changes of pregnancy may be further exacerbated by financial stress, relationship difficulties, and inadequate social support [16,17].

While significant strides have been made over the past decades by most countries, including Zambia, in improving maternal and newborn health outcomes, mental health remains a neglected aspect of maternal healthcare. The lack of comprehensive screening for mental health disorders during ANC visits results in missed opportunities for early detection and intervention [18,19]. Research highlights the potential benefits of targeted screening for common mental disorders, enabling the identification of cases and facilitating timely treatment to mitigate its adverse consequences [18]. However, there is limited evidence on the burden of common mental disorders, particularly among women attending antenatal in Zambia [20]. In poor resource communities such as in Zambia, women attending antenatal care (ANC) services, especially young women, may be at high risk of experiencing mental health challenges [15], but the prevalence and contributing factors of these disorders have not been adequately studied. This study investigated the prevalence of and determinants of common mental disorders among women attending ANC in selected health facilities in Lusaka.

## Methods

### Study design and setting

This was a cross-sectional study aimed to investigate the prevalence of common mental disorders among pregnant women in low-income areas of Lusaka. The study was conducted between 26^th^ April and 6^th^ July 2024 at Kanyama and Matero level one hospitals, situated in suburbs and densely populated peri-urban communities of Lusaka, Zambia. Matero Level one hospital, with a catchment population of about 131,600, has high ANC utilization. Kanyama Level one Hospital, serving a much larger catchment of about 276,000, also provides ANC services. Neither of the two facilities routinely screen for common mental disorders using structured tools. These facilities were purposively selected because of their consistently poor maternal health outcomes such as maternal deaths [21]. Most household heads in these communities are not employed [22]. They depend on small businesses such as selling on the streets and in the market for their income generation. Many households in these areas do not have access to essential infrastructure for electricity, water, sanitation, and effective solid waste management [23,24].

### Study population

#### Inclusion criteria.

The study population was pregnant women aged 15–40 years old attending antenatal care at the selected health facilities in the third trimester as this period provides a more complete picture of ANC utilization, maternal health status, and preparedness for birth.

#### Exclusion criteria.

Pregnant women with pregnancy complications such as hypertensive disorders were excluded from the study to reduce confounding.

### Sample and recruitment of study participants

The sample size for the study was computed using PASS 2023, version 23.0.2. We set out to detect a point estimate (proportion (P)) of 0.263 [25], with a margin of error (e) of 0.05, and a Z value of 1.96 to obtain a 95% confidence level. The calculated minimum number of participants needed for the study was 296. However, the sample size was increased to 338 mothers. This sample size provided approximately 80% power assuming a significance level of 5% to detect an odds ratio of approximately 1.93 or higher for associations between risk factors and common mental disorders. Systematic sampling of every 4th client attending antenatal care was followed until the required sample size was achieved. This method was chosen due to its practicality in high-volume clinical settings and because it does not require a complete sampling frame, making it a feasible and structured approach to participant recruitment [26].

### Data collection

Data for this study was collected using a structured questionnaire. Four trained nurses administered a structured questionnaire to pregnant women in the Maternal and Child Health (MCH) Department of the respective government health facilities. The questionnaire included sociodemographic characteristics, maternal and obstetric factors, the multidimensional scale of perceived social support (MSPSS) and the 20-item Self-reporting questionnaire (SRQ-20). Eligible participants were informed about the study using the research information sheet. Upon agreeing to take part in our study, participants signed consent forms.

### Maternal mental health screening

The 20-item Self-reporting questionnaire (SRQ-20) by the World Health Organization [27] was used to screen the pregnant women for common mental disorders. The SRQ-20 is a CMD screening instrument, which includes 20 yes/ no questions on depression, anxiety, and somatic symptoms as well as suicide ideation experienced in the last 30 days. The tool was used because it is validated for detecting common mental disorders in developing countries [28,29] including Zambia, is cost effective, brief and feasible for busy antenatal clinics. Each item is scored 1 if the answer is “yes”, or 0 if the answer is “no”; therefore, the possible maximum score for the SRQ-20 scale is 20 [27]. The SRQ-20 score was further changed into a binary variable. Under this study, women who scored >= 8 were considered a positive screen for common mental disorders [30].

To assess social connectedness, the MSPSS tool was used to assess perceived adequacy of social connectedness on three dimensions: family, friends, and significant others [31,32]. The MSPSS consists of 12 items that assess perceived social support from family, friends, and significant others. Each item is rated on a 7-point Likert scale ranging from 1 (Very strongly disagree) to 7 (Very strongly agree). A higher score indicated a greater level of perceived social connectedness. Participants were grouped into low (12–35), medium (36–60), and high (61–84) perceived social connectedness categories based on their scores [31].

### Data analysis

All analyses were performed using Stata 18 SE (Stata Corp, College Station, TX, USA). Descriptive statistics such as frequencies and percentages were used to summarize categorical variables, while median and interquartile ranges were used to summarize continuous variables. The chi square, fishers exact and Wilcoxon rank sum test (based on the assumptions of each test) were used to compare common mental disorders across the different characteristics of the women. Logistic regression models were used to identify determinants of common mental disorder. We focused on HIV and the independent variables that were associated with CMD according to the mentioned tests. Directed Acyclic Graphs (DAGs) were used to identify possible confounders to adjust for. Odds ratios and 95% confidence intervals are presented.

### Ethical considerations

Ethical approval was sought from the University of Zambia Biomedical Research Ethics Committee (UNZABREC- Ref-5068–2024). Permission was also sought from relevant authorities such as the National Health Research Authority (NHRA), Ministry of Health and the Lusaka District Health Office. Informed written consent and assent were sought from the study participants and guardians of participants that were below the age of 18 years.

## Results

### Socio-demographic and clinical characteristics of participants

A total of 338 pregnant women were invited to participate in the study, with a 98% response rate. Half of the participants (50.5%) were aged 20–29 years. The majority (69.2%) were married or cohabiting, and nearly three quarters (72.5%) had attained secondary education or higher. About a quarter (23.9%) were living with HIV. Regarding social support, most participants (74.0%) had a medium level of social connectedness, while only 18.4% reported a high level of social connectedness. More than half of the women (58.6%) reported that their current pregnancy was unplanned. Most participants had no previous pregnancy complications (78.0%) and no history of stillbirth (86.7%) or unsafe abortion (87.0%). The majority reported experiencing unpleasant body changes during pregnancy (73.1%) and expressed concern about the delivery process (68.6%) (Table 1).

**Table 1:** Descriptive statistics of study participants.

Characteristic	Frequency n (331)	Percentage %
**Age group**		
< 20 years	42	12.7
20–29 years	167	50.5
≥ 30 years	122	36.9
**Relationship status with the father of child**		
Not in a relationship	43	13.0
In a relationship/ not married	59	17.8
Married/cohabiting	229	69.2
**Education level**		
None	17	5.1
Primary	74	22.4
Secondary or higher	240	72.5
**Employment status**		
Not employed/home maker/housewife	216	65.3
Informal employment/Self-employed/business lady	93	28.1
Formal employment	22	6.6
**Communication with responsible partner**		
No	50	15.1
Yes	281	84.9
**Financial provision**		
Guardian	67	20.2
Others	3	0.9
Self	31	9.4
Spouse	230	69.5
**Current alcohol use**		
No	258	77.9
Yes	73	22.1
**Frequency of having a meal in a day**		
Once	167	50.5
Twice	64	19.3
More than twice	100	30.2
**HIV status**		
HIV -	252	76.1
HIV +	79	23.9
**Social Connectedness**		
Low	25	7.6
Medium	245	74.0
High	61	18.4
**Existing chronic conditions**		
No	242	73.1
Yes	89	26.9
**Current pregnancy planned**		
No	194	58.6
Yes	137	41.4
**History of complications in previous pregnancy (n = 241)**		
No	188	78.0
Yes	53	22.0
**History of stillbirth**		
No	287	86.7
Yes	44	13.3
**History of unsafe abortion**		
No	288	87.0
Yes	43	13.0
**Experience of unpleasant body changes due to current pregnancy**		
No	89	26.9
Yes	242	73.1
**Worried about birth/delivery process**		
No	104	31.4
Yes	227	68.6

n Sample, % Percentage.

### Prevalence of common mental disorders

The overall prevalence of common mental disorders among study participants was 65.8% (218/331; 95% CI 60.5%, 71.0%). The proportion of pregnant women with a common mental disorder was significantly higher among those less than 20 years of age (83.3%; 35/42) as compared to older women aged 20–29 years old (65.9%; 108/164) and women aged 30 years and above (61.5%; 75/122). Furthermore, the proportion with common mental disorders was also significantly higher in women who experienced unpleasant body changes due to the pregnancy (78.3%; 188/240) and women who were worried about the birth/delivery process (78%; 177/227) than those who did not. However, the prevalence of common mental disorders did not substantially differ by HIV status, marital status or employment status, relationship with father of child, alcohol consumption or provider of finances (Table 2).

**Table 2:** Maternal mental health disorder by socio-demographic, clinical and obstetric characteristics of patients.

Characteristic	Number of participants	Maternal mental health disorder		p - value
		Negative screen n (%)	Positive screen n (%)	
**Overall**	328	110 (33.2)	218 (65.8)	
**Age group**				0.034^1^
< 20 years	42	7 (16.7)	35 (83.3)	
20–29 years	164	56 (34.1)	108 (65.9)	
≥30 years	122	47 (38.5)	75 (61.5)	
**Relationship status with the father of child**				0.215^1^
Not in a relationship	43	11 (25.6)	32 (74.4)	
In a relationship but not married	58	16 (27.6)	42 (72.4)	
Married/cohabiting	227	83 (36.6)	144 (63.4)	
**Education level**				0.036^1^
None	17	9 (52.9)	8 (47.1)	
Primary	73	17 (23.3)	56 (76.7)	
Secondary or higher	238	84 (35.3)	154 (64.7)	
**Employment status**				0.827^1^
Not employed/home maker/housewife	215	73 (34.0)	142 (66.0)	
Informal employment/Self-employed/business	92	29 (31.5)	63 (68.5)	
Formal employment	21	8 (38.1)	13 (61.9)	
**Communication with responsible partner**				0.565^1^
No	50	15 (30.0)	35 (70.0)	
Yes	278	95 (34.2)	183 (65.8)	
**Provider of finances**				0.461^1^
Guardian	67	19 (28.4)	48 (71.6)	
Self	31	9 (29.0)	22 (71.0)	
Spouse	230	82 (35.7)	148 (64.3)	
**Current alcohol use**				0.276^1^
No	256	82 (32.0)	174 (68.0)	
Yes	72	28 (38.9)	44 (61.1)	
**Frequency of having a meal in a day**				0.001^1^
once	167	49 (29.3)	118 (70.7)	
twice	63	34 (54.0)	29 (46.0)	
more than twice	98	27 (27.6)	71 (72.4)	
**HIV status**				0.890^1^
HIV -	249	83 (33.3)	166 (66.7)	
HIV +	79	27 (34.2)	52 (65.8)	
**Social Connectedness**				<0.001^2^
Low	25	1 (4.0)	24 (96.0)	
Medium	242	80 (33.1)	162 (66.9)	
High	61	29 (47.5)	32 (52.5)	
**Chronic conditions**				0.236^1^
No	240	76 (31.7)	164 (68.3)	
Yes	88	34 (38.6)	54 (61.4)	
**Number of children (Median/IQR)**	1.0 (0.0 - 2.0)	1.0 (1.0 - 2.0)	1.0 (0.0 - 2.0)	0.093^3^
**Number of pregnancies (Median/IQR)**	2.0 (1.0 - 4.0)	2.5 (2.0 - 3.0)	2.0 (1.0 - 4.0)	0.284^3^
**Systolic blood pressure (Median/IQR)**	117.4 (12.1)	117.2 (12.0)	117.5 (12.2)	0.860^3^
**Diastolic blood pressure (Median/IQR)**	74.0 (70.0 - 80.0)	75.0 (70.0 - 80.0)	74.0 (68.0 - 79.0)	0.162^3^
**Current pregnancy planned**				0.021^1^
No	193	55 (28.5)	138 (71.5)	
Yes	135	55 (40.7)	80 (59.3)	
**History of complications in previous pregnancy**				0.009^1^
No	186	79 (42.5)	107 (57.5)	
Yes	53	12 (22.6)	41 (77.4)	
**History of stillbirth**				0.547^1^
No	284	97 (34.2)	187 (65.8)	
Yes	44	13 (29.5)	31 (70.5)	
**History of unsafe abortion**				0.371^1^
No	285	93 (32.6)	192 (67.4)	
Yes	43	17 (39.5)	26 (60.5)	
**Experience of unpleasant body changes due to current pregnancy**				<0.001^1^
No	88	58 (65.9)	30 (34.1)	
Yes	240	52 (21.7)	188 (78.3)	
**Worried about birth/delivery process**				<0.001^1^
No	104	61 (58.7)	43 (41.3)	
Yes	227	50 (22.0)	177 (78.0)	

HIV Human immunodeficiency virus, n Frequency, % Percentage, ^1^ Chi square test, ^2^ Fisher’s exact test, ^3^ Wilcoxon rank sum test, IQR Interquartile range.

### Determinants of common mental disorders

The adjusted prevalence ratios (adjusted for potential confounders) also indicated that young age, primary level education, unpleasant body changes, having history of complications in previous pregnancy and being worried about the delivery process were particularly strong risk factors for common mental disorders (Table 3). Social connectedness was a strong protective factor.

**Table 3:** Determinants of common mental disorder among pregnant women attending antenatal services.

Characteristics	Unadjusted	Adjusted
**Age**		
< 20 years	3.13 (1.29, 7.63)*	3.04 (1.23, 7.48)*
20–29 years	1.21 (0.74, 1.97)	1.25 (0.76, 2.06)
>=30 years	Ref	Ref
**Education level**		
None	Ref	Ref
Primary	3.70 (1.24, 11.09)*	3.32 (1.09, 10.18)*
Secondary +	2.05 (0.76, 5.51)	1.76 (0.63, 4.87)
**HIV status**		
HIV-	Ref	Ref
HIV+	0.96 (0.56,1.64)	1.16 (0.62, 2.17)
**History of complications in previous pregnancy**		
No	Ref	Ref
Yes	2.52 (1.25,5.11)*	2.37 (1.16, 4.82)*
**Social connectedness**		
Low	Ref	Ref
Medium	0.08 (0.01,0.63)*	0.10 (0.01, 0.74)*
High	0.05 (0.01,0.36)*	0.06 (0.01, 0.48)*
**Current pregnancy planned**		
No	Ref	1.42 (0.80, 2.54)
Yes	0.58 (0.36,0.92)*	Ref
**Worried about birth/delivery process**		
No	Ref	Ref
Yes	4.88 (2.96,8.07)*	10.0 (5, 21.95)*
**Experience of unpleasant body changes due to current pregnancy**		
Yes	Ref	Ref
No	7.18 (4.20, 12.28)*	7.68 (4.36, 13.51)*

OR Odds ratio, CI confidence interval, % Percentage, HIV Human immunodeficiency virus, Ref Reference, * Statistically significant at 5% significance level.

Age group was adjusted for education level. HIV status was adjusted for education and employment status, while education level was adjusted for age group. History of complications in the previous pregnancy was adjusted for number of pregnancies and age group. Social connectedness was adjusted for age group, education level, employment status and HIV status. Current pregnancy planned was adjusted for employment status, history of alcohol drinking, history of unsafe abortions, education level, and history of complications in previous pregnancy. Worried about birth/delivery process was adjusted for age group, education level, employment status, history of complications in previous pregnancy, and financial provision. Experience of unpleasant body changes due to pregnancy was adjusted for age group, education level, number of children, and current pregnancy planned.

## Discussion

This study indicates a relatively high prevalence of common mental disorders (65.8%) among women attending antenatal care at the two selected clinics in low-resource communities of Lusaka, Zambia. Adolescent girls and young women, those with primary education, those who reported complications in a previous pregnancy, those that reported experiences of unpleasant body changes and women worried about the birth/delivery process had higher likelihood of common mental disorders. On the other hand, we found that women who had high level of social connectedness were less likely to have common mental health disorders. The burden of common mental disorders identified in this study is higher compared to what has been found among pregnant women in most other studies in low resource settings such urban Kenya (55%) [33], India (21.9%) [34] and Ethiopia (37.5%) [35]. Although our findings align with previous research on pregnant women in Turkey (64.5%) (17) and Northeast Brazil (63.6%) (18), it is possible that the high estimate can be attributed to the poor living conditions in the two unplanned densely populated communities in this study [23]. Women experiencing socioeconomic disadvantages often face multiple social stressors that contribute to mental health challenges [36,37].

The increased likelihood of common mental disorders among adolescents and young women found in this study is in line with other studies among pregnant adolescents and women [15,38]. Pregnant adolescents and young women may lack the psychological resilience and coping skills needed to manage the physical, emotional, and social transitions that accompany pregnancy and early parenthood [39]. Evidence shows that they are at heightened risk of family rejection, stigma, and social isolation, and may even experience both psychological and social denial of the pregnancy due to fear, pressure, or lack of support [40,41]. In contrast, older pregnant women are more likely to have higher socioeconomic status, stronger social support networks, and more stable relationships factors that offer protection against mental health challenges during pregnancy [42]. Our findings of high burden of CMD among adolescents and young women in this study highlight the critical need for tailored, age-appropriate health services targeting pregnant adolescents and young women by providing contraception to avoid unwanted pregnancies and strengthening social support, such as supported peer groups for young pregnant women [15]. Notably, general access to mental health services is crucial and need to be strengthened.

Women with primary level education had increased odds of common mental health disorders as compared to those with no formal education. However, other studies have reported lesser odds of CMDs among pregnant women with primary education compared with those without formal education [43,44]. Our findings could be attributed to the small sample of participants without formal education and may just reflect random variation.

Similarly, women that had a history of complications in a previous pregnancy as well as women who were worried about the birth/delivery process had higher odds of CMDs. These findings are similar to other studies that have reported anxiety during pregnancy to be attributable to previous complications or difficulty in delivery, specifically caesarean section or instrumental deliveries [45–47]. In contexts with high maternal death rates and uncertain access to quality obstetric care, anxiety associated with delivery may be especially prominent [48]. These findings indicate that comprehensive antenatal care must encompass not just physical health assessment but also psychological support, and education on birth preparedness and requirements for childbirth.

The protective effect of high social connectedness found in this study has been reported elsewhere [17] and represents one of the strongest predictors in this study, emphasizing the crucial role of social support in maternal mental health. This finding aligns with extensive literature demonstrating that strong social networks, family support, and community connections serve as protective factors against common mental disorders [49]. Social support has been shown to enhance life satisfaction and significantly reduces the risk of depression and anxiety during pregnancy [17,50]. However, in most contexts, traditional support systems for pregnant women have been weakened by urbanization, migration, and changing family structures [51]. The low social connectedness observed among pregnant women in urban high-density areas of Lusaka suggests widespread social isolation, likely linked to rapid urbanization, poverty, and limited social and community-supporting infrastructure in informal settlements such as community centers, safe recreational areas, and gathering points [52,53].

The strong association between unpleasant body changes during pregnancy and common health disorders in this study supports the important interconnection between physical and psychological well-being during pregnancy [54]. Pregnancy-related physical changes such as weight gain, breast enlargement, and abdominal growth can significantly impact body image, self-esteem, and overall psychological adjustment [55]. This finding suggests the need for ensuring that prenatal education includes coping strategies for potential psychological and physical changes as they may be particularly distressing and poorly understood.

### Limitations

Some limitations should be considered when interpreting these findings. The cross-sectional design limits our ability to establish causal relationships between identified factors and common mental health disorders. Additionally, the use of self-reported measures for mental health assessment, while validated, may be subject to social desirability bias or cultural factors that influence symptom reporting. However, this study employed a validated tool to assess mental health outcomes and social connectivity, ensuring methodological rigor. Furthermore, this research addresses a significant gap in maternal mental health literature from Zambian contexts, providing important baseline data that can inform future longitudinal studies and intervention development in similar low-resource settings.

## Conclusion

This study reveals a significant burden of maternal mental disorders among women attending antenatal care in high-density areas of Lusaka, Zambia. The finding highlights the need to integrate mental health screening and support into routine antenatal care services. Special attention should be given to younger mothers, those with multiple pregnancies, and women expressing anxiety about childbirth or distress from body changes. Strengthening social support networks can help reduce psychological distress.

## Supporting information

S1 Data Common Mental Disorders among pregnant women – Dataset. (XLS)

S1 Checklist Inclusivity in global health questionnaire. (DOCX)
